# Liquid–Liquid and Vapor–Liquid–Liquid Equilibria of the Alkyl Palmitate + Alkyl–OH + Glycerol Systems at 101.3 kPa—Measurements, Quality Test/Consistency, Thermodynamic Modeling and Molecular Dynamics Simulations

**DOI:** 10.3390/molecules31040604

**Published:** 2026-02-09

**Authors:** Franklin Carvalho, Matheus Pena, Maria Silveira, Nian Freire, Daniela Guimarães, Rima Biswas, Pedro Arce

**Affiliations:** 1 Chemical Engineering Department, Engineering School of Lorena (EEL/USP), University of Sao Paulo, Lorena 12602-810, SP, Brazil; franklindcarv@usp.br (F.C.); mmrpena@usp.br (M.P.); mcarolbs@usp.br (M.S.); nian.freire@hotmail.com (N.F.); dhguima@usp.br (D.G.); 2 School of Chemical Engineering, Vellore Institute of Technology, Vellore 632014, Tamil Nadu, India; rima.biswas@vit.ac.in

**Keywords:** biodiesel, glycerol, multiphase equilibria, NRTL, molecular dynamics simulation

## Abstract

Biodiesel is a biofuel commonly produced through transesterification, also known as alcoholysis. In this process, triglycerides react with short-chain alcohols (alkyl–OH), producing a mixture of fatty acid esters and glycerol. These esters and glycerol are only partially miscible, leading to the formation of two liquid phases during product separation. Therefore, it is important to experimentally determine liquid–liquid (LLE) and/or vapor–liquid–liquid equilibrium (VLLE) data to better understand the transesterification process and to support improvements in reaction rate, selectivity, reactor and mixture simulation, optimization, and separation processes. This work aimed to experimentally measure and thermodynamically model the LLE and VLLE of alkyl palmitate + alkyl–OH + glycerol systems at 101.3 kPa. For the LLE at 318.15 K, the binodal curve was determined, and tie-line compositions were measured in a jacketed equilibrium cell. These data were subjected to quality tests and used to calculate separation factors. For the VLLE, calibration curves were constructed, and experimental data were obtained in a modified Othmer ebulliometer and subsequently tested for consistency. Thermodynamic modeling was performed using γ–γ (LLE) and γ–γ–φ (VLLE) approaches with the Non-Random Two-Liquid (NRTL) activity coefficient model. The experimental and modeling results were analyzed using phase diagrams (triangular and 3D prism representations) and showed that it is possible to clearly separate the palmitate-rich and glycerol-rich liquid phases. In the VLLE, it was observed that the alkyl–OH is essentially pure in the vapor phase. For both types of equilibria, deviations in liquid-phase compositions (LLE), bubble temperatures, and vapor-phase compositions were below 2.0%, indicating that the NRTL model is capable of accurately describing the phase behavior of these systems. The phase equilibria of the methyl/ethyl palmitate–methanol/ethanol–glycerol system were studied using molecular dynamics (MD). The analyses based on the radial distribution function (RDF), spatial distribution function (SDF) and interaction energies showed that methanol and ethanol interact more strongly with glycerol than with palmitates. As a result, the glycerol-rich phase contains more methanol or ethanol, which can significantly reduce costs in the biodiesel purification step.

## 1. Introduction

Global energy demand has increased sharply since the onset of industrialization and is expected to continue rising in the coming decades, which intensifies dependence on fossil fuels and exacerbates environmental impacts such as greenhouse gas emissions and climate change [1,2]. The most widely used fuels are still derived from finite and polluting fossil reserves, such as petroleum, whose price volatility and geopolitical concentration can trigger energy crises, especially in importing countries. In this context, the search for alternative fuels produced from renewable and widely available resources, such as biofuels, has intensified since the late twentieth century [3,4,5].

Biodiesel is technically defined as an alkyl ester (methyl, ethyl, among others) of fatty acids, predominantly obtained by transesterification of oils or fats of vegetable or animal origin with short-chain alcohols, usually methanol or ethanol [3,6]. In the conventional route, vegetable oils are transesterified by heating the mixture with an excess of anhydrous alcohol in the presence of an acid or base catalyst, with alkali hydroxides (NaOH, KOH) or metal alkoxides being the most commonly employed homogeneous catalysts due to their high conversion and low cost [7,8,9]. In this reaction, a high alcohol:oil molar ratio is used to shift the equilibrium and favor the formation of monoalkyl esters (FAME/FAEE), with glycerol as the main byproduct [7].

After the transesterification step, phase separation occurs as follows: a light phase is formed, rich in fatty acid esters and containing excess alcohol, and a heavy phase, predominantly rich in glycerol, together with impurities and traces of catalyst. Owing to the limited miscibility among esters, glycerol, and alcohol, the system exhibits biphasic behavior (or triphasic when vapor is present), making the characterization of liquid–liquid equilibrium (LLE) and vapor–liquid–liquid equilibrium (VLLE) data essential to understand the process, optimize reaction kinetics and selectivity, and design and simulate separation and purification steps. Classical and recent studies on ternary systems composed of methanol + glycerol + methyl oleate [10,11], FAME/FAEE + alcohol + glycerol [12,13,14], and, more recently, systems involving biodiesel from waste oils and different alcohols such as methanol and ethanol [15,16,17] report phase-separation behavior similar to that described here, with the formation of an ester-rich phase and a glycerol-rich phase, and reinforce the importance of using thermodynamic models such as NRTL, UNIQUAC, UNIFAC and their variants for the correlation and prediction of these equilibria at the process scale.

In the study of complex systems, such as these two ternary systems, it is important to emphasize the effect of the polar component on the phase behavior in greater detail, specifically examining its impact on critical points and the stability of the homogeneous phase. The introduction of a polar component is known to significantly influence the thermodynamic properties of the system, altering the position of the critical consolute point and potentially stabilizing the homogeneous phase under certain conditions. This effect is investigated through both experimental data and theoretical models. Additionally, integrating these findings with current phase equilibria models to deepen our understanding of critical-type behavior in ternary systems, particularly focusing on the transition between phase separation and homogeneity in mixtures with varying levels of polarity.

Recent studies suggest that polar components alter the mixing behavior and phase boundaries in both binary and ternary mixtures, with significant shifts in the critical point depending on the nature of interactions. This aligns with findings in phase diagrams where the introduction of polar components causes a shift in consolute temperatures, impacting the miscibility gap and leading to a more homogeneous mixture at different compositions and temperatures [18,19]. Furthermore, recent work highlights how the addition of polar compounds can stabilize or destabilize the homogeneous phase depending on the specific nature of the polar interactions within the mixture [20,21].

Although the present work is based on experimental measurements and activity coefficient modeling (γ–γ and γ–γ–φ approaches with the NRTL model) for the LLE and VLLE of alkyl palmitate + alkyl–OH + glycerol systems at 101.3 kPa, its macroscopic phase behavior is consistent with trends reported in recent molecular dynamics (MD) studies for related biodiesel mixtures [22,23,24]. MD simulations of methyl ester + water systems have shown the formation of an ester-rich phase and a hydrogen-bonded polar phase, together with liquid–liquid and vapor–liquid interfacial properties that agree well with experimental phase diagrams [22]. Likewise, MD studies of triglyceride/methanol mixtures and triolein/cosolvent binary systems highlight the limited miscibility between long-chain lipids and short-chain alcohols and the key role of polar cosolvents in promoting single-phase behavior [23,24]. These microscopic insights are in line with our experimental observation of two liquid phases—an alkyl palmitate–rich phase and a glycerol-rich phase—and with the preferential segregation of the most volatile component (alkyl–OH) into the vapor phase in VLLE. To date, however, there have been no MD studies explicitly addressing the full ternary alkyl palmitate + short-chain alcohol + glycerol system at near-atmospheric pressure, so the LLE and VLLE data reported here provide a useful benchmark for future force field development and validation in biodiesel-related MD simulations.

The aim of this work was to experimentally analyze the phase behavior, represented by the liquid–liquid equilibrium (LLE) and liquid–liquid–vapor equilibrium (VLLE), of the systems alkyl palmitate + alkyl–OH + glycerol at 101.3 kPa (alkyl group: CH_3_ and C_2_H_5_). For the LLE at 333.15 K, an equilibrium cell consisting of a jacketed flask was used, and for the VLLE, a modified Othmer ebulliometer was employed. The experimental data were subjected to a quality test (LLE) and a thermodynamic consistency test (VLLE). The data considered to be of good quality or thermodynamically consistent were correlated with the Non-Random Two-Liquid (NRTL) model. The accuracy of the modeling was evaluated from the relative deviations in composition (LLE) and in bubble temperature and vapor-phase composition (for the VLLE). To comprehend the phase equilibria of the methyl palmitate/ethyl palmitate -methanol/ethanol–glycerol ternary system by examining their structural properties, a molecular dynamics simulation was carried out. The RDFs, SDFs, and interaction energies indicate that methanol or ethanol molecules strongly interact with glycerol as compared to palmitate molecules. These results suggest that the glycerol-rich phase had a higher methanol or ethanol content, which led to significant savings in the biodiesel purification stage.

## 2. Results and Discussions

### 2.1. Experimental Data

#### 2.1.1. Binodal Curve and Calibration Curves

The binodal curves (LLE) of the alkyl palmitate + alkyl–OH + glycerol systems, as well as the calibration curves, are presented in the Supplementary Material. In Table S1, the experimental data for the binodal curves (methyl palmitate and glycerol phases) are shown of the methyl palmitate (1) + methanol (2) + glycerol (3) systems at 333.15 K and 101.3 kPa). These data were obtained at the cloud point. In this table, the refractive index and density are also shown, with which the calibration curves were constructed (Table S2). The same methodology was applied to determine the binodal curve of the ethyl palmitate + ethanol + glycerol system (Table S3) and its calibration curves (Table S4).

In the case of the VLLE, there are no binodal curves. Therefore, samples of different concentrations (molar fractions) were prepared, and the refractive index and density (Tables S5 and S7) were measured at 318.15 K for the methyl palmitate + methanol + glycerol and ethyl palmitate + ethanol + glycerol systems, respectively. With this information, calibration curves were constructed for both ternary systems, and their coefficients are presented in Tables S6 and S8.

#### 2.1.2. LLE Experimental Data

For the LLE, with the help of calibration curves, the equilibrium phase compositions were determined. The experimental data for the LLE (tie-lines) of the alkyl palmitate + alkyl–OH + glycerol systems, measured at 333.15 K and 101.3 kPa, are presented in Table 1 (x represents mole fractions).

#### 2.1.3. VLLE Experimental Data

The multiphase equilibrium compositions of both ternary systems were obtained by knowing the refractive indices and densities of the three phases involved and the corresponding calibration curves. The experimental data measured for the VLLE of the alkyl palmitate + alkyl–OH + glycerol systems, at 101.3 kPa, are presented in Table 2 (x and y represent mole fractions).

It is important to emphasize that only alcohol was found in the vapor phase. This was verified by measuring the refractive index and density of the vapor phase. For the methyl palmitate + methanol + glycerol system, the refractive index and density of the vapor phase were 1.3280 and 0.7681 g·cm^−3^, respectively, while for the ethyl palmitate + ethanol + glycerol system, the values found for the vapor phase were 1.3415 and 0.7531 g·cm^−3^, respectively. These values represent the refractive index and density of methanol and ethanol.

### 2.2. Quality of the Measured LLE and VLLE Experimental Data

#### 2.2.1. Quality Tests

LLE experimental data were subjected to the quality tests. Results of the analysis by the method proposed by Marcilla and collaborators [25] appear in Table 3 for the experimental data at 333.15 K and 101.3 kPa for the alkyl palmitate + alkyl–OH + glycerol systems at 333.15 K and 101.3 kPa. This same table also shows the compositions (mole fractions) of the solutions that gave rise to the tie-lines.

It can be observed that the largest deviations found were 0.06% and 0.30% for the tie-lines for the methyl palmitate + methanol + glycerol and ethyl palmitate + ethanol + glycerol systems, respectively, at a temperature of 333.15 K. However, the average deviation among all tie-lines was 0.0230% and 0.0825% for both ternary systems. Thus, all experimental data meet the criterion of deviation of less than 0.5%, verifying the quality of the LLE experimental data, ensures the reliability of the results for the next step, which is thermodynamic modeling.

#### 2.2.2. Consistency Tests

VLLE experimental data were subjected to the consistency tests. Results of the analysis by the method proposed by Wisniak [26] appear in Table 4 for the experimental data at 101.3 kPa for the alkyl palmitate + alkyl–OH + glycerol systems.

This table also shows that for the methyl palmitate + methanol + glycerol system, all measured VLLE data had deviations of less than 0.81%, while for the ethyl palmitate + ethanol + glycerol system, the maximum deviation was 0.73%. Knowing that D must be less than 3.0%, VLLE experimental data for both systems are considered thermodynamically consistent. Average deviations were 0.3722% and 0.6073% for the methyl palmitate + methanol + glycerol and ethyl palmitate + ethanol + glycerol systems, respectively.

The saturation pressures for methyl palmitate, ethyl palmitate, methanol, ethanol, and glycerol used to analyze the thermodynamic consistency test were calculated based on the Antoine equation, and the coefficients of this equation [27,28,29] are presented in the Supplementary Material (Table S9).

### 2.3. Separation Factor (S) and Distribution Coefficient (D) for the LLE

LLE experimental separation factors and distribution coefficients were calculated using data validated by the quality test. Separation factor (S) and distribution coefficients (D) for both ternary systems, at a temperature of 333.15 K, are available in Table 5. Analyses were first performed by assuming palmitates as solvents and glycerol as diluent, and second, palmitates as diluents and glycerol as solvent, for both cases, alcohol always as solute. Numerical values for these analyses are shown in Table 5.

Comparing the distribution coefficient and mainly the separation factor (S) for methanol or ethanol, presented in Table 5, it can be concluded that glycerol would behave better as a solvent than as a diluent for both ternary systems. This is because the separation factors are greater than 1.0.

### 2.4. Thermodynamic Modeling

Typically, the study of thermodynamic modeling of LLE and the VLLE behavior is carried out using the gamma–gamma (γ-γ) and the gamma–gamma–phi (γ-γ-ϕ) approaches. The activity coefficients of the components of both liquid phases, γ, were obtained from the NRTL (Non-Random Two-Liquid) [30] thermodynamic model, and because the pressure is 101.3 kPa, the fugacity coefficients were assumed to have a value of 1.0.

To obtain the NRTL parameters for the LLE and VLLE, an optimization process based on the Levenberg–Marquardt algorithm [31] was used, minimizing the following Objective Functions (OFs) (Equations (1) and (2)), based on the γ–γ and γ–γ–ϕ approaches [32,33].

(1) $$OF=\frac{1}{n}\sum \left|{x_{i}}^{palm}{\gamma_{i}}^{palm}-{x_{i}}^{gly}{\gamma_{i}}^{gly}\right|$$

(2) $$OF=\frac{1}{n}\sum \left|{x_{i}}^{palm}{P_{i}}^{SAT}{\gamma_{i}}^{palm}-y_{i}P{{\hat{\Phi }}_{i}}^{VAP}\right|+\frac{1}{n}\sum \left|{x_{i}}^{gly}{P_{i}}^{SAT}{\gamma_{i}}^{gly}-y_{i}P{{\hat{\Phi }}_{i}}^{VAP}\right|$$

For the LLE, the binary interaction parameters of the NRTL model are presented in Table 6, while the randomness parameter was kept constant (α_ij_ = 0.3).

The absolute deviations for the compositions of both liquid phases, for the two ternary systems, were obtained with the NRTL model, using the gamma–gamma (γ-γ) approach, at 101.3 kPa. At temperatures of 333.15, the mean square deviations (RMSDs) were 0.0024 and 0.0038 for the NRTL model, for the methyl palmitate + methanol + glycerol and ethyl palmitate + ethanol + glycerol systems, respectively.

For the VLLE, the binary interaction parameters of the NRTL model are presented in Table 7, while the randomness parameter was kept constant (α_ij_ = 0.3).

The mean absolute deviations for bubble temperature and vapor phase compositions (alcohol and glycerol), obtained with the NRTL model and using the γ-γ-ϕ approach, at 101.3 kPa, are 0.71, 0.11, and 0.11 and 1.02, 0.09, and 0.09, for the methyl palmitate + methanol + glycerol and ethyl palmitate + ethanol + glycerol systems, respectively. Deviations for temperature and compositions were obtained using Equations (3) and (4).

(3) $$\begin{array}{c} ∆T\left(\%\right)=\frac{\left|T_{exp}-T_{,calc}\right|}{T_{exp}}*100 \end{array}$$

(4) $$\begin{array}{c} ∆y_{i}\left(\%\right)=\left|y_{i,exp}-y_{i,calc}\right|*100 \end{array}$$

**Figure 1:**
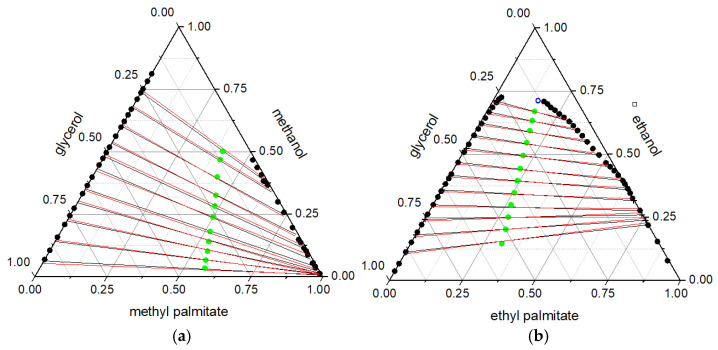
LLE phase diagrams of alkyl palmitate + alkyl–OH + glycerol systems at 333.15 K (101.3 kPa). (**a**) Methyl palmitate + methanol + glycerol system. (**b**) Ethyl palmitate + ethanol + glycerol system.

The LLE phase diagrams of the alkyl palmitate + alkyl–OH + glycerol system are presented in Figure 1, (a) for the methyl palmitate + methanol + glycerol system and (b) for the ethyl palmitate + ethanol + glycerol system. The phase diagrams contain the binodal curve (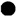), the experimental tie-lines (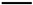), and the tie-lines obtained through thermodynamic modeling (NRTL: 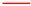) at 333.15 K. The total compositions of the solutions (feed solutions) that gave rise to the tie-lines (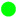) are also shown in this figure, as well as the predicted crit-ical point (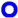). The predicted critical point of the ethyl palmitate (1) + ethanol (2) + glycerol (3) system was determined using the Alders method [34], and its compositions are: x_1_ = 0.1548, x_2_ = 0.7119, x_3_ = 0.1333. For the methyl palmitate + methanol + glycerol system, the critical point could not be predicted. It is also important to emphasize that the phase behavior in the ELL of both ternary systems is of type I.

**Figure 2:**
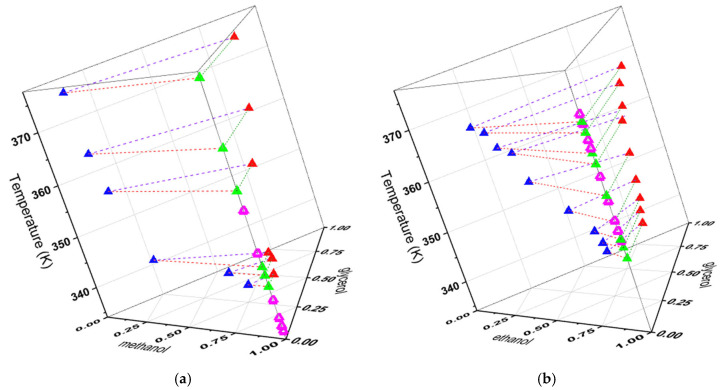
VLLE diagrams of alkyl palmitate + alkyl–OH + glycerol systems at 101.3 kPa. (**a**) Methyl palmitate + methanol + glycerol system. (**b**) Ethyl palmitate + ethanol + glycerol system.

Figure 2 shows the following measured VLLE experimental data of the alkyl palmitate + alkyl–OH + glycerol systems: (a) methyl palmitate + methanol + glycerol system, (b) ethyl palmitate + ethanol + glycerol system. The multiphase equilibrium, involving the two liquid phases and the vapor phase, is represented by the following symbols: Experimental palmitate phase: 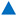; Experimental glycerol phase: 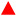; Experimental vapor phase: 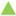; Calculated vapor phase (NRTL): 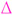. The bubble curves are: palmitate phase (L1-V: 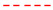), glycerol phase (L2-V: 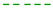), tie lines (L1-L2: 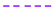). The bubble temperatures obtained with the NRTL model are very similar, numerically, to those obtained experimentally.

In the LLE and VLLE phase diagrams, it is possible to observe that the NRTL model was able to correlate the data from the alkyl palmitate + alkyl–OH + glycerol systems. This is confirmed by the deviations, described above, indicating the NRTL model is appropriate for systems containing complex components present in biodiesel production.

### 2.5. MD Simulation Analysis

Four distinct systems (LLE1, LLE2, VLLE1, and VLLE2) were simulated at 333.15 K. Molecular analysis will begin by discussing the molecular structures of solvents and solutes near the interface. The density profiles and structural properties of the solvents are also investigated.

#### 2.5.1. Molecular Structures at the Interface for LLE and VLLE Systems

**Figure 3:**
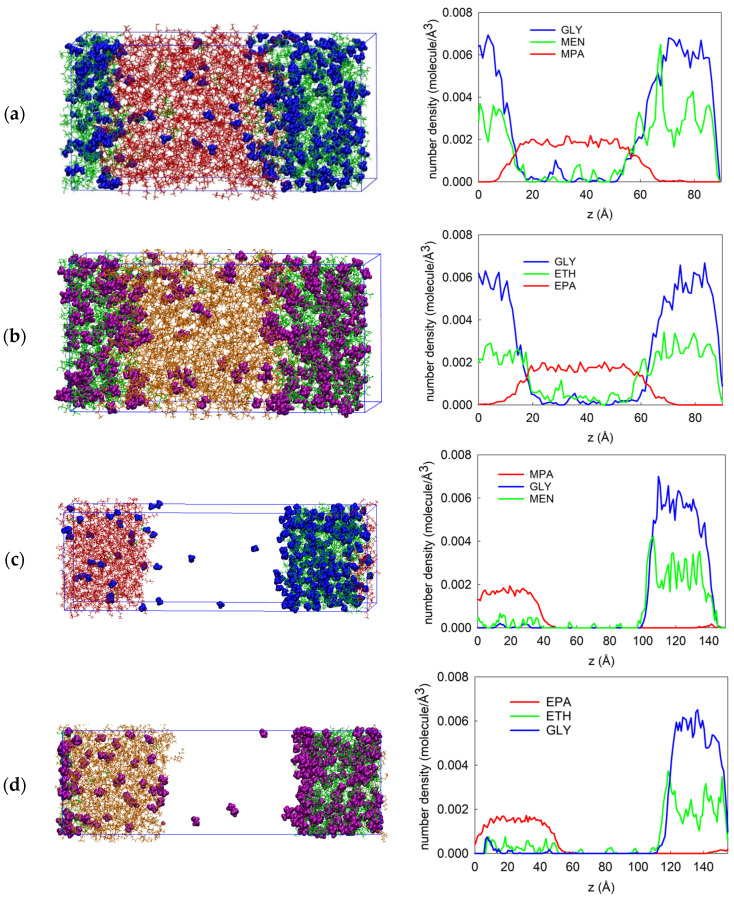
Final images of (**a**) LLE1 [glycerol (GLY)—green, methanol (ME)—blue, methyl palmitate (MPA)—red], (**b**) LLE2 [glycerol (GLY)—green, ethanol (ETH)—purple, ethyl palmitate (EPA)—orange], (**c**) VLLE1[glycerol (GLY)—green, methanol (MEN)—blue, methyl palmitate (MPA)—red], and (**d**) VLLE2 [glycerol (GLY)—green, ethanol (ETH)—purple, ethyl palmitate (EPA)—orange] systems. The number of density profiles for all systems is attached to the right side.

Figure 3 describes how the LLE and VLLE systems are configured. The phases are first separated for the LLE-based system, and methanol or ethanol molecules are added to both solvent phases. It was discovered that some methanol molecules are present in the bulk methyl palmitate phase and the interface after the end of the simulation for the LLE1 system. Similarly, some ethanol molecules migrated in the bulk ethyl palmitate phase and the interface for LLE2 systems. We investigated the phenomenon by calculating the density profiles of solvents and solutes, as seen in Figure 3. The density peak of methanol or ethanol molecules in the glycerol phase was found to be higher than that of the palmitate phase, which suggests that a lower amount of methanol or ethanol dissolves in the palmitate phase and a higher concentration of methanol or ethanol in the glycerol phase. We discovered that ethanol molecules had a higher density in the bulk ethyl palmitate phase (Figure 3a), while methanol molecules had a lower density in the methyl palmitate phase (Figure 3b). It indicates that ethanol molecules are fairly aggregated in the ethyl palmitate phase and exhibit interactions with ethyl palmitate molecules, as evidenced by the higher density of ethanol in the bulk ethyl palmitate phase. Figure 3a,b show the methyl palmitate and ethyl palmitate density distributions over an area z = 5.4–70.2 Å. These results imply that methyl palmitate and ethyl palmitate molecules prefer to remain at the interface and in the bulk palmitate phase.

As seen in Figure 3c, the vapor phase of the VLLE-based system is first positioned between two liquid phases. The methanol and ethanol molecules were observed to diffuse from the liquid phase to the vapor phase during the simulation. We found that methanol and ethanol had a maximum density from 50 Å to 90 Å in the vapor phase (Figure 3c,d). Additionally, there is a correlation between the density profile and the mole percentage of methanol or ethanol in the vapor phase. On the other hand, the glycerol and methyl or ethyl palmitate molecules are absent from the vapor phase, which is proportional to the mole fraction of glycerol and palmitate molecules in the vapor.

As stated in Table 8, the density of each component in the solvent phase enables the calculation of the mole fraction in each phase, where x and y represent the liquid phase and vapor phase mole fractions. Each component’s MD-predicted mole fraction and the experimental value agree quite well.

#### 2.5.2. Structural Properties of Solvents

**Figure 4:**
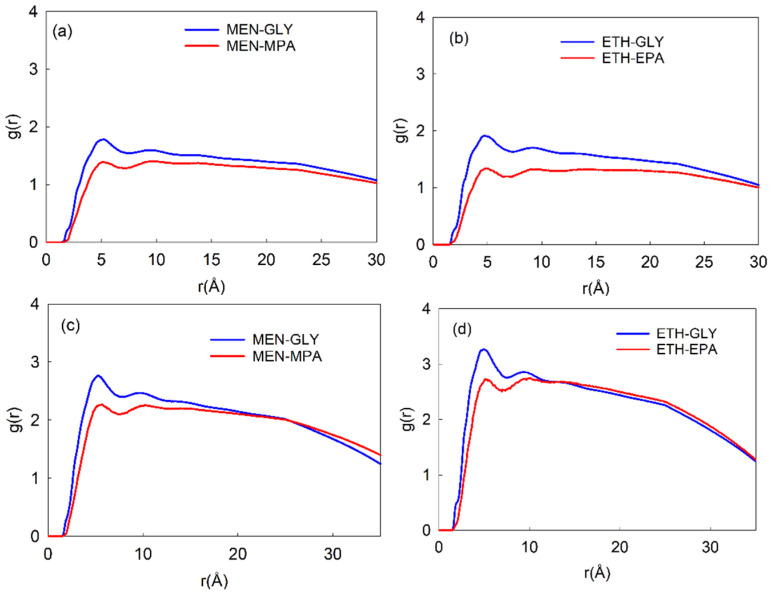
Radial distribution functions of (**a**) methanol (MEN) with glycerol (GLY) and methanol (MEN) with methyl palmitate (MPA) in LLE1, (**b**) ethanol (ETH) with glycerol (GLY) and ethanol (ETH) with ethyl palmitate (EPA) in LLE2, (**c**) methanol (MEN) with glycerol (GLY) and methanol (MEN) with methyl palmitate (MPA) in VLLE1, and (**d**) ethanol (ETH) with glycerol (GLY) and ethanol (ETH) with ethyl palmitate (EPA) in VLLE2 systems.

**Figure 5:**
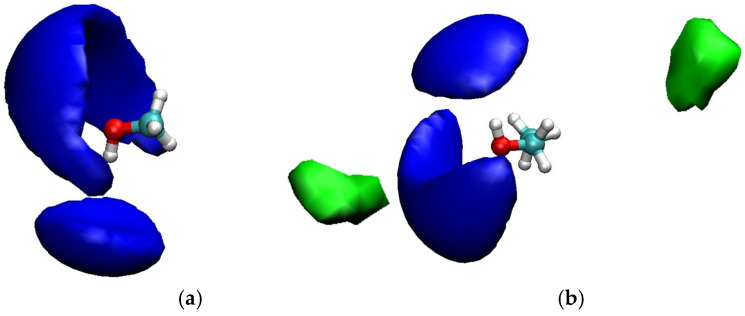
Spatial distribution functions of (**a**) glycerol (blue) and methyl palmitate (green) around methanol and (**b**) glycerol (blue) and ethyl palmitate (green) around ethanol.

The average radial packing of atoms near one another is described by the radial distribution function (RDF), a pair correlation function. VMD, a molecular visualization tool, was used to calculate the RDF plots (Figure 4) [35]. RDFs of methanol with glycerol and methyl palmitate and ethanol with glycerol and ethyl palmitate for LLE and VLLE systems are displayed in Figure 4. The RDF peak height of methanol/ethanol with glycerol was found to be higher as compared to the methyl/ethyl palmitate, indicating the strong interaction between methanol/ethanol and glycerol. The peak value of RDF for ethanol with glycerol (r = 4.92 Å) and ethyl palmitate (r = 5.25 Å) was found to be higher than that of methanol in LLE and VLLE systems. It implies that ethanol molecules have a strong interaction with glycerol and ethyl palmitate molecules when compared with methanol. The spatial distribution function (SDF) of glycerol and palmitate molecules around methanol or ethanol molecules further supports the findings as mentioned in Figure 5. The distribution of glycerol around the active side of the methanol or ethanol was found to be higher than that of palmitate molecules, as confirmed by the SDF calculations. These findings show that glycerol interacts with ethanol or methanol molecules more than palmitate molecules, which is consistent with the RDF plots.

We have calculated the interaction energies by adding the following two contributions: electrostatic and van der Waals (vdW) interaction, as indicated in Table 9. The interaction energy between methanol and glycerol is higher (−16.233 kJ/mol and −13.267 kJ/mol) than that of methyl palmitate (−3.540 kJ/mol and −3.535 kJ/mol) in LLE1 and VLLE1 systems. A further close investigation reveals that the interaction energies of ethanol–glycerol (−17.402 kJ/mol and −13.613 kJ/mol) and ethanol–ethyl palmitate (−3.879 kJ/mol and −3.773 kJ/mol) are higher than those of methanol–glycerol and methyl–palmitate in LLE2 and VLLE2 systems. Additionally, it should be mentioned that there is less solubility due to the reduced interaction energy between glycerol and palmitate molecules, which leads to bigger phase separations.

## 3. Materials and Methods

### 3.1. Chemicals

All reagents, presented in Table 10, were subjected to a drying process and degassed under vacuum at room temperature for several days before being used. At this stage, the physical properties of the reagents, refractive index, and density were measured. The physical properties, refractive index (ηD) and density (ρ) of the reagents used are presented in Table 1 (for methyl palmitate, methanol, and glycerol at 298.15 K and for ethyl palmitate and ethanol at 333.15 K).

Physical properties were measured using the DMA 1001 densimeter with an accuracy of ±0.0001 g·cm^−3^, and the Abbemat 3200 refractometer with an accuracy of ±0.0001, both from Anton Paar (Graz, Austria).

### 3.2. Experimental Equipment

#### 3.2.1. LLE Equipment

**Figure 6:**
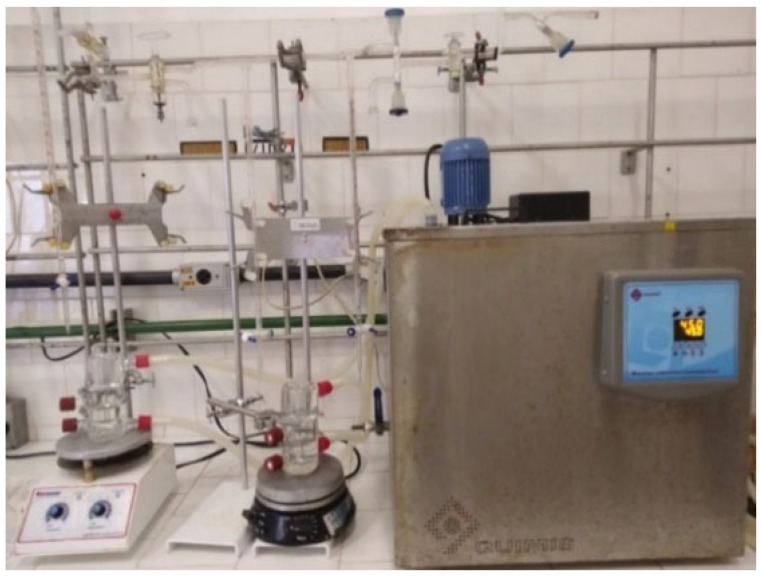
Equipment for the LLE analysis.

The LLE was studied in a transparent jacketed flask made of borosilicate glass, allowing visualization of the phases present in the system. A thermostatic bath was coupled to the cell to maintain a constant temperature (333.15 K). The cell has side collectors, through which samples can be removed without disturbing the phase equilibrium, and two Teflon caps to seal them when not in use. The assembled equipment is shown in Figure 6 [42,43,44].

#### 3.2.2. LLE and VLLE Experimental Equipment

**Figure 7:**
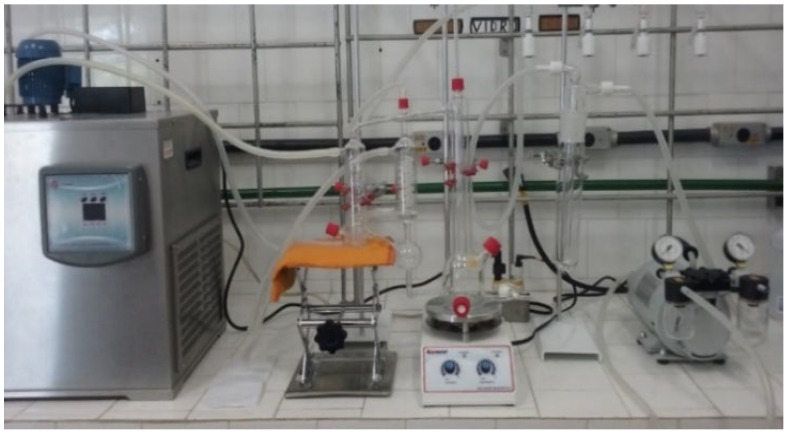
Equipment for the VLLE analysis.

The VLLE was studied in a modified Othmer ebulliometer made of borosilicate glass (Figure 7). The load is inserted into the ebulliometer reservoir, and heat is supplied by a heating/magnetic plate. When the liquid solution begins to boil, the vapor rises up the column and goes to the condenser, maintained at 288.15 K. As a result, the vapor phase condenses and returns in droplets to the ebulliometer reservoir. The validity of the experimental equipment used in the VLLE analysis has already been tested in previous studies [28,45].

Samples of each phase are taken from the equilibrium cell and the ebulliometer after equilibrium has been reached, and then the refractive indices and densities are measured and recorded. These properties are used to determine the compositions of the two liquid phases (LLE) and the two liquid phases and the vapor phase (VLLE), using calibration curves.

### 3.3. Experimental Procedure

#### 3.3.1. Binodal Curve (LLE) and Calibration Curves (LLE and VLLE) of Alkyl Palmitate + Alkyl–OH + Glycerol Systems

The binodal curve for the LLE was determined experimentally using the titration method in a jacketed glass vessel connected to a thermostatic bath (Model TE, TECNAL, Piracicaba, Brazil) with a temperature uncertainty of 0.1 K. The solution was continuously stirred at a temperature of 308.15 K and a pressure of 101.3 kPa, similar to previous studies [40,41]. The biodiesel-rich phase was titrated with glycerol by gradually adding known quantities, while the glycerol phase was titrated with the biodiesel until it reached a characteristic turbidity, known as the cloud point, and the solution was stirred for thirty minutes. This process was repeated multiple times with different compositions to map the binodal curve.

Using the refractive indices and densities measured for the binodal curve (LLE) at the cloud point, calibration curves were implemented for the LLE. For the VLLE, solutions of known compositions were prepared, the refractive index and density of each phase were measured, and the calibration curves were implemented. With the refractive index and density data as a function of composition, for the LLE and VLLE, it was possible to establish second-order polynomial curves (calibration curves), such as those presented in Equations (5) and (6).

(5) $$\eta_{D}\left(x_{1},x_{2}\right)=A_{00}+A_{10}x_{1}+A_{01}x_{2}+A_{20}x_{1}^{2}+A_{11}x_{1}x_{2}+A_{02}x_{2}^{2}$$

(6) $$\rho \left(x_{1},x_{2}\right)={A’}_{00}+{A’}_{10}x_{1}+{A’}_{01}x_{2}+{A’}_{20}x_{1}^{2}+{A’}_{11}x_{1}x_{2}+{A’}_{02}x_{2}^{2}$$

These calibration curves are used to determine the compositions in the LLE and VLLE, knowing the refractive index and density of the phases. It is important to note that the composition of the third component, x_3_, is obtained only by knowing that the sum of the compositions must be 1.0.

#### 3.3.2. Measurement of the Multiphase Experimental Data of Alkyl Palmitate + Alkyl–OH + Glycerol Systems

##### Measurements of the LLE Data

**Figure 8:**
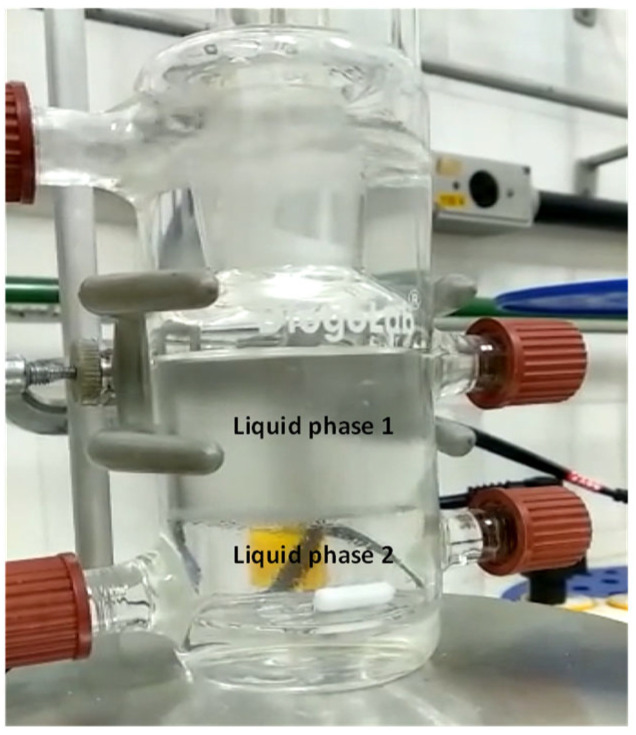
Phase separation in the LLE experimental analysis.

After determining the binodal curve, LLE tie-lines were acquired by maintaining constant temperature and stirring for twenty minutes, then allowing the solution to rest at a constant temperature for twenty-four hours to form two liquid phases. Afterward, samples from each phase were collected, and their refractive indices and densities were measured in triplicate. These properties were used to calculate the compositions of each phase using the binodal curve as a calibration tool. The compositions of both phases provided the LLE tie-line (Figure 8).

##### Measurements of the VLLE Data

The modified Othmer ebulliometer, made of borosilicate glass, was used to study the VLLE behavior of the mixture. The system pressure was kept constant using a Type 2 VC pump (Model 121, PRISMATEC, Itu, Brazil) with a tolerance of 1.0 kPa. Heat was supplied to the equilibrium cell by a magnetic heater and stirrer, while the vapor phase was cooled by two condensers at 288.15 K using an ultra-thermostatic bath with external circulation (QUIMIS Model Q214m2, Diadema, Brazil). The temperatures of the two liquid and vapor phases were measured using Kanagroo Traceable and GM1312 thermometers (Hampton, NH, USA), respectively, with an accuracy of 0.1 K.

**Figure 9:**
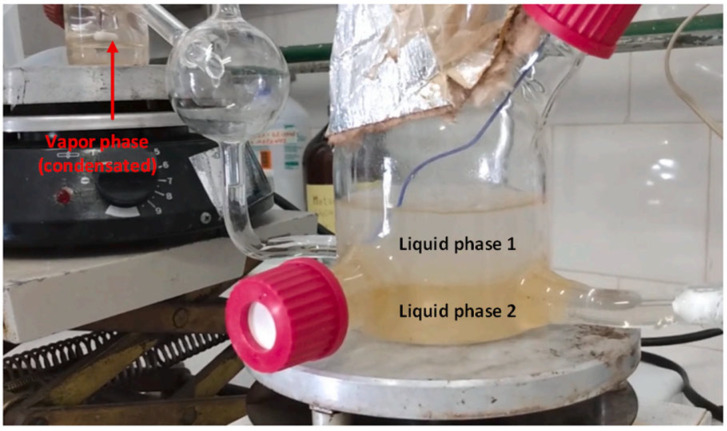
Phase separation in the VLLE experimental analysis.

Once the mixture was introduced into the equilibrium cell, heating and stirring began. As the mixture vaporized, a condensed vapor phase filled its respective cell and returned to the equilibrium cell in drops. After the system reached equilibrium, indicated by a constant temperature at the vapor–liquid–liquid interface for over an hour, samples from the two liquid and vapor phases were carefully collected. The refractive index and density of the three phases were measured, and their compositions were determined using calibration curves, previously constructed. This procedure and equipment are similar to those used in prior studies [28,44]. Figure 9 illustrates the three phases in the VLLE after equilibrium is reached, showing the two liquid phases at the front and the condensed vapor phase at the bottom.

### 3.4. Quality of the Multiphase Experimental Data

Researchers study the quality of liquid–liquid equilibrium (LLE) and vapor-liquid–liquid equilibria (VLLE) experimental data for several important reasons, related to both the reliability and application of this data in various industrial and scientific contexts. There are many reasons to do that, such as the accuracy of thermodynamic models, optimization of industrial processes, design of separation units, prediction of phase behavior, development of new solvents or reactions, consistency and reproducibility, regulatory compliance, and advanced applications and research.

#### 3.4.1. Quality Test (LLE)

The method applied is based on the mass balance test to ensure that the mass is conserved in the extraction process. For a given component, compare the mass of the component before and after the extraction, where the mass before extraction is equal to mass in feed plus mass in solvent, and the mass after extraction is equal to mass in extract (L1) plus mass in raffinate (L2). If there is a discrepancy (i.e., a significant deviation), it could indicate an error in measurement, loss of material during transfer, or evaporation [25].

According to the method proposed by Marcilla and collaborators [25], experimental LLE data are evaluated based on mass balance. The sum of the calculated mass in both phases of equilibrium is compared with the actual value of the total experimental mass (M^sol^). They also established that the good quality of the experimentally obtained data is guaranteed if the overall deviations of the mass balance, δ, are less than 0.5%. Equations (7)–(9) indicate the calculation of the total mass balance of each component:

(7) $$\begin{array}{c} M^{sol}x_{1}^{sol}=M^{PAL}x_{1}^{PAL}+M^{GLY}x_{1}^{GLY} \end{array}$$

(8) $$\begin{array}{c} M^{sol}x_{2}^{sol}=M^{PAL}x_{2}^{PAL}+M^{GLY}x_{2}^{GLY} \end{array}$$

(9) $$\begin{array}{c} M^{sol}x_{3}^{sol}=M^{PAL}x_{3}^{PAL}+M^{GLY}x_{3}^{GLY} \end{array}$$

where $x_{1}^{PAL},\;x_{2}^{PAL},\;x_{3}^{PAL},\;x_{1}^{GLY},\;x_{2}^{GLY}\;and\;x_{3}^{GLY}\;$ represent the compositions of each of the components (1: methyl palmitate or ethyl palmitate, 2: methanol or ethanol, 3: glycerol) of both liquid phases (PAL and GLY) in equilibrium. The set of linear algebraic equations can be represented in matrix form, as shown in Equation (10).

(10) $$\begin{array}{c} \underset{M}{\underbrace{\left[\begin{array}{cc} M^{sol} & x_{1}^{sol}\\ M^{sol} & x_{2}^{sol}\\ M^{sol} & x_{3}^{sol} \end{array}\right]}}=\underset{B}{\underbrace{\left[\begin{array}{cc} x_{1}^{PAL} & x_{1}^{GLY}\\ x_{2}^{PAL} & x_{2}^{GLY}\\ x_{3}^{PAL} & x_{3}^{GLY} \end{array}\right]}}.\underset{P}{\underbrace{\left[\begin{array}{c} M^{PAL}\\ M^{GLY} \end{array}\right]}} \end{array}$$

Explicitly defining vector P, it appears as

(11) $$\begin{array}{c} P={\left(B^{T}B\right)}^{-1}B^{T}M \end{array}$$

where B^T^ is the transpose of matrix B and X^−1^ is the inverse of X. By numerically solving Equation (11), the values of M^PAL^ and M^GLY^ can be obtained, and the sum of M^PAL^ and M^GLY^ can be compared with the value of M^sol^. The overall deviation of the mass balance, δ, can be obtained by Equation (12).

(12) $$\begin{array}{c} \delta \left(\%\right)=100\cdot \frac{\left|\left(M^{PAL}+M^{GLY}\right)-M^{sol}\right|}{M^{sol}} \end{array}$$

#### 3.4.2. Consistency Tests (VLLE)

The Wisniak’s consistency tests [26] are a well-established set of criteria used to evaluate the reliability and thermodynamic consistency of vapor–liquid–liquid (VLLE) equilibrium data. These tests, developed by Wisniak and collaborators [26], are widely used in phase equilibrium studies to assess the consistency of experimental data with respect to the thermodynamic principles governing phase equilibria.

The goal of thermodynamic consistency is to allow the thermodynamic model to exactly fit the experimental data, respecting the Gibbs–Duhem equation criterion. The thermodynamic consistency test can be applied to experimental phase equilibrium data for binary and ternary systems. The L-W test [26] is one of the most important methods used in VLE and VLLE for binary or ternary systems; it uses the excess Gibbs free energy of the system. In this method, two parameters, L_i_ and W_i_, are calculated, as shown in Equations (13) and (14).

(13) $$L_{i}=\frac{\sum T_{k}^{SAT}x_{k}∆S_{k}^{SAT}}{\sum x_{k}∆S_{k}^{SAT}}-T$$

(14) $$W_{i}=\frac{RT}{\sum x_{k}∆S_{k}^{SAT}}\left[\sum x_{k}ln\gamma_{k}-\sum x_{k}\ln\frac{\gamma_{k}}{x_{k}}\right]$$

where $T_{k}^{SAT}$ and D$S_{k}^{SAT}$ are the saturation temperature and saturation entropy of the k component, respectively. These two parameters are used to calculate L and W (Equation (15)), and they must be similar. The difference between the two values is used to calculate the deviation D (Equation (16)). If the value of D is less than 3.0%, the system will be considered thermodynamically consistent.

(15) $$L=\int_{0}^{1}L_{i}{d_{x}}_{1}=\int_{0}^{1}W_{i}{d_{x}}_{1}=W$$

(16) $$D=100\frac{\left|L-W\right|}{L+W}$$

### 3.5. Separation Factor (S) and Distribution Coefficient (D) for the LLE

Before thermodynamic modeling of the LLE, analyzing experimental data regarding the solvent’s ability to extract the solute from the diluent is a relevant concept in industrial processes to verify the effectiveness of separation in aqueous or organic solutions [43]. For this, the distribution coefficients of the diluent, D_1_, and of the solute, D_2_, are used, according to Equations (17) and (18), in that order, to calculate the separation factor or selectivity [42,43].

(17) $$D_{1}=\frac{x_{1}^{phase\;3}}{x_{1}^{phase\;1}}$$

(18) $$D_{2}=\frac{x_{2}^{phase\;3}}{x_{2}^{phase\;1}}$$

where x_1_ and x_2_ represent the mole fractions of the diluent and solute, respectively, and the superscripts phase 1 and phase 3 refer to the diluent-rich and solvent-rich phases.

The separation factor, S, is defined as the quotient between these solute and diluent distribution coefficients (Equation (19)).

(19) $$S=\frac{D_{2}}{D_{1}}$$

For effective separation to occur, the goal is for the values of S to be greater than 1.0.

### 3.6. Thermodynamic Modeling

#### 3.6.1. Phase Equilibria

The thermodynamic behavior of LLE and the VLLE is based on the equality of pressures, temperatures, and chemical potentials or fugacities for each component in both phases. If we designate the liquid phases as PAL and GLY and the vapor phase as V, the LLE and the VLLE criteria for a system of N components [26], at constant pressure and temperature, are shown in Equations (21) and (22) for the LLE and VLLE, respectively.

(20) $${\hat{f}}_{i}^{PAL}={\hat{f}}_{i}^{GLY}$$

(21) $${\hat{f}}_{i}^{PAL}={\hat{f}}_{i}^{GLY}={\hat{f}}_{i}^{V}$$

In terms of activity coefficients and fugacity coefficients, Equations (20) and (21) can be represented as Equations (22) and (23).

(22) $$x_{i}^{PAL}\gamma_{i}^{PAL}=x_{i}^{GLY}\gamma_{i}^{GLY}$$

(23) $$x_{i}^{PAL}P_{i}^{sat,PAL}\gamma_{i}^{PAL}=x_{i}^{GLY}P_{i}^{sat,GLY}\gamma_{i}^{GLY}=y_{i}{\hat{\emptyset }}_{i}^{V}P$$

In this research work, the pressure was kept constant at 101.3 kPa. Thus, the fugacity coefficients have a value of 1.0.

#### 3.6.2. Non-Random Two Liquid (NRTL) Model

The activity coefficient was calculated using the NRTL model, developed by Renon and Prausnitz [30]. This model provides parameters for calculating the activity coefficient of a solute in a liquid solvent in the study of partially miscible or completely miscible non-ideal systems. Activity coefficients can be determined from Equation (24).

(24) $$\ln\gamma_{i}=\frac{\sum_{j}\tau_{ji}G_{ji}x_{j}}{\sum_{k}G_{ki}x_{k}}+\sum_{j}\frac{x_{j}G_{ij}}{\sum_{k}G_{kj}x_{k}}\left(\tau_{ij}-\frac{\sum_{r}x_{r}\tau_{rj}G_{rj}}{\sum_{k}G_{kj}x_{k}}\right)$$

where

(25) $$\tau_{ij}=\frac{g_{ij}-g_{jj}}{RT}$$

(26) $$G_{ij}=\exp\left(-\alpha_{ij}\tau_{ij}\right)$$

The parameters $g_{ij}$ and $\alpha_{ij}$ equate the characteristic energy of the i-j interactions and the non-randomness of the system, respectively. Non-randomness is the foundation of the NRTL model, which considers that the components in solution follow a distribution pattern according to the local composition.

#### 3.6.3. LLE and VLLE Deviations

For the LLE, one way to determine the efficiency of thermodynamic models is to calculate the root mean square deviation (RMSD) (Equation (27)).

(27) $$RMSD=100\sum_{k}^{N}\sum_{j}^{2}\sum_{i}^{3}\frac{{\left(x_{ijk}^{exp}-x_{ijk}^{calc}\right)}^{2}}{6N}$$

where i, j and k represent the component, phase and the number of tie-lines, respectively, x is the mole fraction, “exp” and “calc” correspond to the experimental data and the one calculated by the NRTL model.

For the VLLE, the bubble point temperature and the vapor phase composition are taken into account. Using the experimental bubble point temperatures and those calculated by the NRTL model, the relative deviation is calculated, and using the molar fractions in the vapor phase, the absolute deviation is calculated.

### 3.7. Molecular Dynamics Simulation

Gauss View 05 [46] was used to create the structures and geometries of the methanol, ethanol, methyl palmitate, ethyl palmitate, and glycerol. Gaussian 09 [47] optimized these structures at the B3LYP/6-31G* level of theory. The LigParGen Server [48] was used to produce all the force field parameters needed to execute MD simulation in accordance with the generalized OPLSS-AA force field [49]. The generated force field parameters are confirmed by comparing the simulated density of methanol (0.7841 g·cm^−3^), ethanol (0.7248 g·cm^−3^), methyl palmitate (0.8531 g·cm^−3^), ethyl palmitate (0.8074 g·cm^−3^), and glycerol (1.1164 g·cm^−3^) under ambient condition with those of the experimental density values (0.8080 g·cm^−3^, 0.7538 g·cm^−3^, 0.8960 g·cm^−3^, 0.8298 g·cm^−3^, and 1.2349 g·cm^−3^).

The initial configurations of LLE1 and VLLE1 systems consisted of methyl palmitate, methanol and glycerol, whereas LLE2 and VLLE2 systems consisted of ethyl palmitate, ethanol and glycerol. The initial configurations of the LLE systems were first arranged in a 45 Å × 45 Å × 90 Å box using PACKMOL version 18.169 [49]. The number of molecules in each of the four systems for which MD simulations were performed is listed in Table 11, where x and n are the mole fraction and mole number, respectively. Initially, two different compartments were filled with methyl palmitate or ethyl palmitate and glycerol molecules for LLE systems. The boxes were then positioned in close proximity to one another in order to simulate or generate a two-phase system, that is, a palmitate-rich phase and a glycerol-rich phase. The methanol or ethanol molecules were then evenly dispersed throughout both phases. The NAnoscale Molecular Dynamics (NAMD) software version 2.14 [50] used Langevin thermostat and Langevin barostat [51] to run all of the MD simulations. The initial size of the periodic box was set as 50 Å × 50 Å × 155 Å for VLLE systems. A time integration step of 1 fs was used for all simulations. The LLE-simulated systems were first minimized for 1 ns, and then they were gradually heated for 1 ns from 0 to 333.15 K. The VLLE1 system was heated from 0 to 365.65 K, and for VLLE2, the system was heated from 0 to 362.61 K. After the heating process, all systems were then equilibrated for 10 ns using an NPT ensemble at 101.3 kPa pressure. Following that, each system with the NVT ensemble had a 100 ns production run. A 12 Å atom-based cutoff was used for the non-bonded interactions. Similarly, the Ewald summation approach [52] was used to compute the long-range electrostatic interactions. The bonds containing hydrogen atoms were restrained using the SHAKE algorithm [53]. The periodic boundary conditions were used in all three dimensions to eliminate the edge effect. The analysis was done by the Visual Molecular Dynamics (VMD) tool [35].

## 4. Conclusions

LLE and VLLE data of alkyl palmitate + alkyl–OH + glycerol systems were measured at 101.3 kPa, using equipment validated in previous studies.

For the study of the LLE of methyl palmitate + methanol + glycerol and ethyl palmitate + ethanol + glycerol systems, the cloud point method was used to determine the behavior of the binodal curve at a temperature of 333.15 K (type I). With the binodal curves, it was possible to determine the coefficients of the calibration curves, essential for determining the compositions of the tie-lines (LLE). In addition, the properties that a good solvent should have in LLE processes were analyzed. From the alcohol separation factors, it was determined that glycerol is a more efficient solvent than alkyl palmitate for alcohol extraction. Experimental LLE data were subjected to a quality test, and it was found that all experimental tie-lines met the criteria proposed by Marcilla et al. The gamma–gamma approach and the NRTL model were used in the thermodynamic modeling. Results, in terms of root mean square deviations, indicated that the model based on excess Gibbs free energy is suitable for predicting the LLE behavior of systems containing components in biodiesel production.

For the VLLE, calibration curves were prepared as a function of refractive index and density. Solutions were prepared and introduced into the experimental unit (modified Othmer ebulliometer); taking into account the criteria of thermodynamic equilibrium (constant pressure and temperature) and also the constant dripping rate, equilibrium was reached. The refractive index and density of the two liquid and condensed vapor phases were measured. With the help of the calibration curves, the equilibrium compositions were obtained. These experimental data were subjected to a thermodynamic consistency test, verifying that all were consistent from a thermodynamic point of view. Finally, thermodynamic modeling was performed using the gamma–gamma–phi approach and the NRTL model. The relative mean deviations for the bubble point temperature and absolute deviations for the vapor phase compositions were very satisfactory, with values less than 1.0%, showing that the model is capable of correlating data in the multiphase equilibria of systems containing complex components.

To comprehend the phase equilibria of the methyl palmitate/ethyl palmitate -methanol–/ethanol–glycerol ternary system by examining their structural properties, a molecular dynamics simulation was carried out. The RDFs, SDFs, and interaction energies indicate that methanol or ethanol molecules strongly interact with glycerol as compared to palmitate molecules. These results suggest that the glycerol-rich phase had a higher methanol or ethanol content, which led to significant savings in the biodiesel purification stage.

## Figures and Tables

**Table 1:** Tie-lines of the alkyl palmitate + alkyl–OH + glycerol systems at 333.15 K and 101.3 kPa.

Biodiesel Phase			Glycerol Phase		
x_1_	x_2_	x_3_	x_1_	x_2_	x_3_
Methyl palmitate (1) + methanol (2) + glycerol (3)					
0.6066	0.3835	0.0099	0.0001	0.7382	0.2618
0.6536	0.3381	0.0083	0.0001	0.6778	0.3222
0.7482	0.2447	0.0071	0.0001	0.6027	0.3973
0.8245	0.1678	0.0077	0.0001	0.5341	0.4659
0.8786	0.1123	0.0091	0.0001	0.4816	0.5184
0.9238	0.0690	0.0072	0.0001	0.4325	0.5675
0.9563	0.0354	0.0083	0.0001	0.3511	0.6489
0.9807	0.0123	0.0071	0.0001	0.2961	0.7040
0.9820	0.0121	0.0059	0.0001	0.2231	0.7769
0.9823	0.0122	0.0056	0.0001	0.1403	0.8597
0.9828	0.0121	0.0051	0.0001	0.0626	0.9374
Ethyl palmitate (1) + ethanol (2) + glycerol (3)					
0.3041	0.6305	0.0654	0.0119	0.7103	0.2778
0.3498	0.5939	0.0562	0.0092	0.6667	0.3241
0.4061	0.5480	0.0460	0.0066	0.6221	0.3713
0.4726	0.4959	0.0315	0.0036	0.5742	0.4222
0.5328	0.4496	0.0176	0.0034	0.5229	0.4737
0.6045	0.3951	0.0004	0.0029	0.4673	0.5299
0.6408	0.3587	0.0004	0.0024	0.4156	0.5820
0.6819	0.3158	0.0023	0.0019	0.3582	0.6399
0.7234	0.2757	0.0009	0.0014	0.2989	0.6998
0.7381	0.2594	0.0024	0.0011	0.2418	0.7571
0.7611	0.2376	0.0012	0.0006	0.1798	0.8196
0.7778	0.2197	0.0025	0.0005	0.1117	0.8879

Standard uncertainties: u(T) = 0.01 K. Expanded uncertainty (*p* = 0.95, k = 2): Uc(xi) = 0.0002.

**Table 2:** VLLE experimental data for the alkyl palmitate + alkyl–OH + glycerol systems at 101.3 kPa.

Temperature (K)	Palmitate Phase			Glycerol Phase			Vapor Phase
	x_1_	x_2_	x_3_	x_1_	x_2_	x_3_	y_2_
Methyl palmitate (1) + methanol (2) + glycerol (3)							
343.05	0.0982	0.9016	0.0002	0.0000	0.8995	0.1005	≈1.0000
344.95	0.1771	0.8227	0.0002	0.0000	0.8586	0.1414	≈1.0000
346.35	0.5697	0.4297	0.0005	0.0000	0.8796	0.1204	≈1.0000
358.85	0.6927	0.3071	0.0002	0.0000	0.7298	0.2702	≈1.0000
365.65	0.7302	0.2696	0.0002	0.0000	0.5495	0.4505	≈1.0000
376.55	0.7457	0.2541	0.0002	0.0000	0.4452	0.5548	≈1.0000
Ethyl palmitate (1) + ethanol (2) + glycerol (3)							
346.87	0.1129	0.8393	0.0478	0.0004	0.6801	0.3194	≈1.0000
348.72	0.1117	0.8555	0.0328	0.0002	0.6589	0.3409	≈1.0000
350.14	0.1483	0.7918	0.0600	0.0003	0.6117	0.3880	≈1.0000
353.08	0.2672	0.6389	0.0939	0.0002	0.5886	0.4113	≈1.0000
357.43	0.4633	0.3924	0.1442	0.0003	0.5569	0.4429	≈1.0000
362.61	0.5090	0.3403	0.1507	0.0001	0.5180	0.4819	≈1.0000
364.36	0.5566	0.3391	0.1043	0.0000	0.4552	0.5447	≈1.0000
367.68	0.5800	0.3501	0.0699	0.0002	0.3935	0.6063	≈1.0000
369.47	0.6142	0.3722	0.0136	0.0003	0.2873	0.7124	≈1.0000

Standard uncertainties: u(T) = 0.01 K, u(P) = 1.00 kPa; expanded uncertainty (*p* = 0.95, k = 2): Uc(xi) = 0.0002.

**Table 3:** Quality tests of the alkyl palmitate + alkyl–OH + glycerol systems at 333.15 K and 101.3 kPa.

Feed Solution				M^PAL^	M^GLY^	M^sol^ (cal)	δ
M^sol^ (g)	x_1_	x_2_	x_3_	(g)	(g)	(g)	
Methyl palmitate (1) + methanol (2) + glycerol (3)							
13.4206	0.5742	0.0336	0.3997	7.8415	5.5720	13.4135	0.0526
13.8611	0.5613	0.0658	0.3636	7.9212	5.9355	13.8567	0.0321
13.8132	0.5781	0.0965	0.3146	8.1308	5.6767	13.8075	0.0415
13.8915	0.5677	0.1344	0.3067	8.0418	5.8409	13.8827	0.0632
14.0222	0.5498	0.1726	0.2862	8.0632	5.9576	14.0208	0.0102
14.2907	0.5333	0.2266	0.2492	8.2507	6.0389	14.2896	0.0074
14.3318	0.5126	0.2712	0.2267	8.3616	5.9687	14.3303	0.0103
14.7126	0.4641	0.3225	0.2032	8.2831	6.4285	14.7116	0.0065
14.6241	0.4294	0.4022	0.1766	8.3916	6.2313	14.6229	0.0084
14.8209	0.3731	0.4894	0.1456	8.4605	6.3583	14.8188	0.0141
14.9141	0.3515	0.5396	0.1183	8.6423	6.2708	14.9131	0.0065
Ethyl palmitate (1) + ethanol (2) + glycerol (3)							
8.5236	0.1643	0.6696	0.1661	2.7053	5.8208	8.5261	0.0300
7.9543	0.1744	0.6329	0.1926	2.6747	5.2816	7.9563	0.0200
8.0562	0.1863	0.5928	0.2209	2.8295	5.2270	8.0566	0.0000
10.9654	0.1991	0.5452	0.2557	4.0010	6.9598	10.9608	0.0400
10.6325	0.2123	0.4945	0.2932	4.0084	6.6228	10.6313	0.0100
12.4152	0.2282	0.4425	0.3292	4.7551	7.6522	12.4072	0.0600
9.8415	0.2427	0.3948	0.3625	3.7321	6.1076	9.8397	0.0200
8.6328	0.2561	0.3463	0.3977	3.2496	5.3716	8.6213	0.1300
10.2548	0.2687	0.2989	0.4324	3.8377	6.3862	10.2239	0.3000
9.9418	0.2833	0.2504	0.4663	3.6739	6.2615	9.9355	0.0600
8.6325	0.2990	0.2019	0.4990	3.1408	5.4933	8.6341	0.0200
11.5284	0.3132	0.1457	0.5410	4.1879	7.3747	11.5626	0.3000

**Table 4:** Thermodynamic consistency results for the alkyl palmitate + alkyl–OH + glycerol systems at 101.3 kPa.

L	W	D (%)	Consistency Test
Methyl palmitate (1) + methanol (2) + glycerol (3)			
16.8902	16.9430	0.1602	Consistent
24.4956	24.6117	0.2411	Consistent
34.3605	34.9190	0.8103	Consistent
37.5643	37.6320	0.0905	Consistent
40.3311	40.1314	0.2507	Consistent
34.4727	34.0095	0.6802	Consistent
Ethyl palmitate (1) + ethanol (2) + glycerol (3)			
30.2506	30.5684	0.5225	Consistent
30.7360	31.1218	0.6237	Consistent
35.3653	35.8526	0.6842	Consistent
34.9331	35.2108	0.3959	Consistent
34.4304	34.8854	0.6564	Consistent
34.0003	34.4108	0.6000	Consistent
32.1013	32.5107	0.6336	Consistent
30.6516	31.1041	0.7327	Consistent
30.3685	30.7451	0.6162	Consistent

**Table 5:** Separation factors (S) and distribution coefficients of methanol/ethanol (D2) (T = 333.15 K, P = 101.3 kPa).

D_1_	D_2_	S	D_1_	D_2_	S
Methyl palmitate: solvent; glycerol: diluent; methanol: solute			Methyl palmitate: diluent; glycerol: solvent; methanol: solute		
9828.0000	0.1933	0.0000	0.0001	5.1736	50846
9823.0000	0.0870	0.0000	0.0001	11.5000	112965
9820.0000	0.0542	0.0000	0.0001	18.4380	181061
9807.0000	0.0415	0.0000	0.0001	24.0732	236086
9563.0000	0.1008	0.0000	0.0001	9.9181	94847
9238.0000	0.1595	0.0000	0.0001	6.2681	57905
8786.0000	0.2332	0.0000	0.0001	4.2885	37679
8245.0000	0.3142	0.0000	0.0001	3.1830	26243
7482.0000	0.4060	0.0001	0.0001	2.4630	18428
6536.0000	0.4988	0.0001	0.0002	2.0047	13103
6066.0000	0.5195	0.0001	0.0002	1.9249	11676
Ethyl palmitate: solvent; glycerol: diluent; ethanol: solute			Ethyl palmitate: diluent; glycerol: solvent; ethanol: solute		
25.5546	0.8877	0.0347	0.0391	1.1266	29
38.0217	0.8908	0.0234	0.0263	1.1226	43
61.5303	0.8809	0.0143	0.0163	1.1352	70
131.2778	0.8636	0.0066	0.0076	1.1579	152
156.7059	0.8598	0.0055	0.0064	1.1630	182
208.4483	0.8455	0.0041	0.0048	1.1827	247
267.0000	0.8631	0.0032	0.0037	1.1586	309
358.8947	0.8816	0.0025	0.0028	1.1343	407
516.7143	0.9224	0.0018	0.0019	1.0841	560
671.0000	1.0728	0.0016	0.0015	0.9322	625
1268.5000	1.3215	0.0010	0.0008	0.7567	960
1555.6000	1.9669	0.0013	0.0006	0.5084	791

**Table 6:** NRTL binary interaction parameters, ∆g_ij_ (J·mol^−1^), for the alkyl palmitate + alkyl–OH + glycerol systems at 333.15 K and 101.3 kPa (LLE).

i/j	1	2	3
Methyl palmitate (1) + methanol (2) + glycerol (3)			
1	0.0000	23.7090	39.4196
2	−4.3526	0.0000	2.6461
3	18.8716	−4.3606	0.0000
Ethyl palmitate (1) + ethanol (2) + glycerol (3)			
1	0.0000	27.6911	50.2868
2	−20.2578	0.0000	4.1424
3	27.9800	−6.4686	0.0000

**Table 7:** NRTL binary interaction parameters, ∆g_ij_ (J·mol^−1^), for the alkyl palmitate + alkyl–OH + glycerol systems at 333.15 K and 101.3 kPa (VLLE).

i/j	1	2	3
Methyl palmitate (1) + methanol (2) + glycerol (3)			
1	0.0000	22.6535	36.1442
2	−12.7342	0.0000	7.1364
3	38.5939	−6.3003	0.0000
Ethyl palmitate (1) + ethanol (2) + glycerol (3)			
1	0.0000	28.8002	45.3263
2	−15.6381	0.0000	6.6051
3	36.5282	−7.5898	0.0000

**Table 8:** Experimental and MD predicted LLE and VLLE data for LLE1, LLE2, VLLE1, and VLLE2 systems.

Systems		Palmitate Phase			Glycerol Phase			Vapor Phase		
		x_1_	x_2_	x_3_	x_1_	x_2_	x_3_	y_1_	y_1_	y_3_
LLE1	Exp.	0.982	0.012	0.005	0.000	0.062	0.937	-	-	-
LLE1	MD	0.958	0.032	0.009	0.000	0.048	0.951	-	-	-
LLE2	Exp.	0.777	0.219	0.002	0.000	0.111	0.887	-	-	-
LLE2	MD	0.758	0.236	0.005	0.000	0.174	0.825	-	-	-
VLLE1	Exp.	0.730	0.269	0.000	0.000	0.549	0.450	0.000	≈1.000	0.000
VLLE1	MD	0.641	0.355	0.003	0.000	0.454	0.545	0.000	0.999	0.000
VLLE2	Exp.	0.509	0.340	0.150	0.000	0.518	0.481	0.000	≈1.000	0.000
VLLE2	MD	0.727	0.152	0.120	0.000	0.462	0.537	0.000	0.999	0.000

**Table 9:** The interaction energies (kJ/mol) between the different species in various systems.

Systems	Component	Component	Interaction Energies (kJ/mol)
LLE1	Methanol	Glycerol	−16.233
	Methanol	Methyl palmitate	−3.540
	Methyl palmitate	Glycerol	−1.476
LLE2	Ethanol	Glycerol	−17.402
	Ethanol	Ethyl palmitate	−3.879
	Ethyl palmitate	Glycerol	−1.203
VLLE1	Methanol	Glycerol	−13.267
	Methanol	Methyl palmitate	−3.535
	Methyl palmitate	Glycerol	−1.309
VLLE2	Ethanol	Glycerol	−13.613
	Ethanol	Ethyl palmitate	−3.773
	Ethyl palmitate	Glycerol	−1.189

**Table 10:** Physical properties of the reagents used in this work.

Reagent	Supplier	CAS	Purity (%)	In This Work		Literature	
				η_D_	ρ (g·cm^−3^)	η_D_	ρ (g·cm^−3^)
Methyl palmitate	*	112-39-0	99.5%	1.4302	0.8514	1.4310 ^a^	0.8520 ^a^
Ethyl palmitate	*	628-97-7	99.6%	1.4236	0.8298	1.4212 ^b^	0.8305 ^c^
Methanol	Synth	67-56-1	99.7%	1.3286	0.7688	1.3292 ^d^	0.7699 ^d^
Ethanol	Synth	64-17-5	99.5%	1.3422	0.7538	1.3439 ^e^	0.7550 ^e^
Glycerol	Synth	56-81-5	99.6%	1.4721	1.2306	1.4730 ^f^	1.2613 ^f^

* Chengdu Zhongheng Rujie Trading Co. Ltd. (Chengdu, China); ^a^ [36]; ^b^ [37]; ^c^ [38]; ^d^ [39], ^e^ [40]; ^f^ [41]. u(T) = 0.01 K, u(P) = 1.00 kPa. Combined expanded uncertainties Uc (level of confidence = 0.95, k = 2) are Uc(ρ) = 2 × 10^−4^ g·cm^−3^, Uc(ηD) = 2.0 × 10^−4^.

**Table 11:** Number of molecules for the alkyl palmitate + alkyl–OH + glycerol systems configurations in MD simulations.

methyl palmitate (1) + methanol (2) + glycerol (3)							
	x_1_	x_2_	x_3_	n_1_	n_2_	n_3_	n_T_
LLE1	0.185	0.300	0.515	185	300	515	1000
VLLE1	0.185	0.300	0.515	185	300	515	1000
ethyl palmitate (1) + ethanol (2) + glycerol (3)							
	x_1_	x_2_	x_3_	n_1_	n_2_	n_3_	n_T_
LLE2	0.180	0.300	0.520	180	300	520	1000
VLLE2	0.180	0.300	0.520	180	300	520	1000

## Data Availability

The original contributions presented in this study are included in the article. Further inquiries can be directed to the corresponding author.

## Supplementary Materials

The following supporting information can be downloaded at https://www.mdpi.com/article/10.3390/molecules31040604/s1, Table S1. Binodal curves of the methyl palmitate (1) + methanol (2) + glycerol (3) systems (T = 333.15 K at 101.3 kPa), Table S2. Coefficients of the calibration curves (LLE) for the methyl palmitate + methanol + glycerol system (333.15 K and 101.3 kPa), Table S3. Binodal curves of the ethyl palmitate (1) + ethanol (2) + glycerol (3) systems (T = 333.15 K at 101.3 kPa), Table S4. Coefficients of the calibration curves (LLE) for the ethyl palmitate + ethanol + glycerol system (333.15 K and 101.3 kPa), Table S5. Refractive indices and densities (318.15 K) for the methyl palmitate (1) + methanol (2) + glycerol (3) systems (VLLE), Table S6. Coefficients of the calibration curves (VLLE) for the methyl palmitate + methanol + glycerol system (318.15 K and 101.3 kPa), Table S7. Refractive indices and densities for the ethyl palmitate (1) + ethanol (2) + glycerol (3) system at 318.15 K (VLLE), Table S8. Coefficients of the calibration curves (VLLE) for the ethyl palmitate + ethanol + glycerol system at 318.15 K, Table S9. Constants from the Antoine equation are used to calculate the saturation pressure.
