# Supplementary material for: Liquid–Liquid and Vapor–Liquid–Liquid Equilibria of the Alkyl Palmitate + Alkyl–OH + Glycerol Systems at 101.3 kPa—Measurements, Quality Test/Consistency, Thermodynamic Modeling and Molecular Dynamics Simulations
Source: Molecules. 2026 Feb 9;31(4):604. doi: 10.3390/molecules31040604 (PMC12943323; doi:10.3390/molecules31040604)
Supplement: Supplementary file 1 [file molecules-31-00604-s001.zip › molecules-4110151-supplementary.pdf]

Supplementary material

Liquid–Liquid and Vapor–Liquid–Liquid Equilibria of the Alkyl Palmitate + Alkyl–OH + Glycerol Systems at 101.3 kPa—Measurements, Quality Test/Consistency, Thermodynamic Modeling and Molecular Dynamics Simulations

Franklin Carvalho [1], Matheus Pena [1], Maria Silveira [1], Nian Freire [1], Daniela Guimarães [1], Rima Biswas [2] and Pedro Arce [1,*]

[1] Chemical Engineering Department, Engineering School of Lorena (EEL/USP), University of Sao Paulo, Lorena 12602-810, SP, Brazil; franklindcarv@usp.br (F.C.); mmrpena@usp.br (M.P.); mcarolbs@usp.br (M.S.); nian.freire@hotmail.com (N.F.); dhguima@usp.br (D.G.)

[2] School of Chemical Engineering, Vellore Institute of Technology, Vellore 632014, Tamil Nadu, India; rima.biswas@vit.ac.in

* Correspondence: parce@usp.br; Tel.: +55-12-31595326

Table S1. Binodal curves of the methyl palmitate (1) + methanol (2) + glycerol (3) systems (T = 333.15 K and 101.3 kPa)

Palmitate phase					Glycerol phase				
x_1	x_2	x_3	ηD	ϱ (g.cm^{-3})	x_1	x_2	x_3	ηD	ϱ (g.cm^{-3})
0.9837	0.0118	0.0045	1.4253	0.8969	0.0000	0.0685	0.9315	1.4516	1.2849
0.9832	0.0119	0.0049	1.4252	0.8970	0.0000	0.1048	0.8952	1.4511	1.2820
0.9828	0.0119	0.0053	1.4251	0.8972	0.0000	0.1575	0.8425	1.4387	1.2394
0.9821	0.0121	0.0058	1.4250	0.8974	0.0000	0.2083	0.7917	1.4313	1.2134
0.9817	0.0122	0.0061	1.4250	0.8975	0.0000	0.2438	0.7562	1.4262	1.1952
0.9563	0.0354	0.0083	1.4228	0.8964	0.0000	0.2735	0.7265	1.4218	1.1800
0.9533	0.0403	0.0064	1.4258	0.9300	0.0000	0.3318	0.6682	1.4210	1.1798
0.9393	0.0545	0.0062	1.4272	0.9120	0.0000	0.3646	0.6354	1.4086	1.1333
0.9374	0.0537	0.0089	1.4208	0.8951	0.0000	0.3982	0.6018	1.4037	1.1161
0.9038	0.0874	0.0089	1.4171	0.8921	0.0000	0.4469	0.5531	1.4030	1.1025
0.8918	0.1018	0.0064	1.4226	0.9160	0.0000	0.4733	0.5267	1.3928	1.0777
0.8769	0.1142	0.0089	1.4141	0.8897	0.0000	0.4962	0.5038	1.3895	1.0659
0.8548	0.1361	0.0091	1.4117	0.8879	0.0000	0.5183	0.4817	1.3862	1.0546
0.8416	0.1487	0.0098	1.4157	0.9160	0.0000	0.5626	0.4374	1.3798	1.0319
0.7967	0.1959	0.0075	1.4051	0.8819	0.0000	0.5984	0.4016	1.3766	1.0121
0.7365	0.2567	0.0068	1.3984	0.8763	0.0000	0.6216	0.3784	1.3712	1.0017
0.6942	0.2992	0.0066	1.3937	0.8725	0.0000	0.6479	0.3521	1.3674	0.9883
0.6248	0.3665	0.0087	1.3863	0.8674	0.0000	0.6702	0.3298	1.3625	0.9850
0.6075	0.3828	0.0097	1.3846	0.8664	0.0000	0.7107	0.2893	1.3583	0.9561
0.6030	0.3825	0.0145	1.3964	0.9120	0.0000	0.7363	0.2637	1.3545	0.9430
0.5827	0.4074	0.0099	1.3818	0.8643	0.0000	0.7519	0.2481	1.3523	0.9350
0.5797	0.4203	0.0000	1.3801	0.8590	0.0000	0.7821	0.2179	1.3479	0.9196
0.5536	0.4367	0.0097	1.3786	0.8617	0.0000	0.8117	0.1883	1.3436	0.9044
0.5231	0.4671	0.0098	1.3753	0.8591					
0.3920	0.5967	0.0113	1.3610	0.8483					
0.2380	0.7548	0.0072	1.3435	0.8326					

Table S2. Coefficients of the calibration curves (LLE) for the methyl palmitate + methanol + glycerol system (333.15 K and 101.3 kPa)

Coeficients	ηD	ϱ (g.cm^{-3})	Std error (η_D)	Std error (ϱ)
		Palmitate phase		
A_{00}	1.4311	0.9134	0.0039	0.0017
A_{10}	-0.1205	-0.1307	0.0012	0.0049
A_{01}	-1.2321	-3.9581	0.0076	0.0039
A_{20}	0.0046	0.0264	0.0013	0.0060
A_{11}	0.2678	0.2360	0.0030	0.0075
A_{02}	85.2497	42.4445	0.0693	0.0569
		Glycerol phase		
A_{00}	1.4627	1.3249	0.0021	0.0068
A_{10}	68.8672	8,0495	0.0000	0,0853
A_{01}	-0.1418	-0,5098	0.0011	0.0339
A_{20}	1.0000	1.0000	0.0000	0.0000
A_{11}	1.0000	1.0000	0.0000	0.0000
A_{02}	-0.0071	-0.0115	0.0014	0.0368

Table S3. Binodal curves of the ethyl palmitate (1) + ethanol (2) + glycerol (3) systems (T = 333.15 K and 101.3 kPa)

Palmitate phase					Glycerol phase				
x_1	x_2	x_3	ηD	ρ (g.cm^{-3})	x_1	x_2	x_3	ηD	ρ (g.cm^{-3})
0.1771	0.7082	0.1147	1.3975	0.8490	0.0215	0.7401	0.2384	1.3860	0.9312
0.1941	0.6987	0.1072	1.3987	0.8458	0.0176	0.7183	0.2640	1.3882	0.9473
0.2121	0.6850	0.1029	1.4001	0.8440	0.0151	0.7045	0.2804	1.3896	0.9575
0.2317	0.6743	0.0941	1.4013	0.8407	0.0115	0.6854	0.3030	1.3915	0.9717
0.2540	0.6594	0.0866	1.4027	0.8381	0.0092	0.6669	0.3239	1.3935	0.9841
0.2768	0.6458	0.0773	1.4039	0.8352	0.0079	0.6434	0.3487	1.3959	0.9978
0.3034	0.6292	0.0674	1.4052	0.8324	0.0064	0.6206	0.3730	1.3983	1.0111
0.3257	0.6124	0.0619	1.4064	0.8311	0.0054	0.5999	0.3947	1.4004	1.0225
0.3500	0.5931	0.0568	1.4076	0.8301	0.0037	0.5740	0.4223	1.4029	1.0368
0.3763	0.5730	0.0507	1.4087	0.8290	0.0034	0.5389	0.4578	1.4063	1.0534
0.4048	0.5490	0.0462	1.4100	0.8284	0.0031	0.5077	0.4891	1.4091	1.0674
0.4383	0.5217	0.0400	1.4112	0.8276	0.0028	0.4666	0.5306	1.4127	1.0850
0.4717	0.4969	0.0314	1.4123	0.8263	0.0027	0.4210	0.5763	1.4165	1.1033
0.5117	0.4651	0.0231	1.4135	0.8253	0.0024	0.4034	0.5942	1.4179	1.1104
0.5330	0.4504	0.0166	1.4141	0.8243	0.0022	0.3819	0.6159	1.4196	1.1186
0.5553	0.4342	0.0105	1.4146	0.8235	0.0018	0.3532	0.6450	1.4218	1.1294
0.5777	0.4163	0.0060	1.4152	0.8231	0.0014	0.3305	0.6681	1.4235	1.1379
0.6057	0.3937	0.0006	1.4159	0.8227	0.0012	0.2994	0.6994	1.4257	1.1486
0.6183	0.3817	0.0000	1.4162	0.8229	0.0011	0.2617	0.7373	1.4283	1.1611
0.6367	0.3633	0.0000	1.4167	0.8234	0.0009	0.2203	0.7787	1.4310	1.1741
0.6542	0.3456	0.0002	1.4172	0.8239	0.0007	0.1909	0.8084	1.4329	1.1833
0.6730	0.3247	0.0022	1.4177	0.8247	0.0006	0.1503	0.8491	1.4353	1.1950
0.7223	0.2767	0.0009	1.4189	0.8255	0.0005	0.1122	0.8873	1.4375	1.2056
0.7794	0.2185	0.0020	1.4201	0.8268	0.0004	0.0660	0.9336	1.4401	1.2179
0.8438	0.1541	0.0021	1.4213	0.8279	0.0003	0.0361	0.9635	1.4417	1.2256
0.9166	0.0776	0.0058	1.4225	0.8296					

Table S4. Coefficients of the calibration curves (LLE) for the ethyl palmitate + ethanol + glycerol system (333.15 K and 101.3 kPa)

Coeficients	η_D	ϱ (g.cm^{-3})	Std error (η_D)	Std error (ϱ)
$\boldsymbol{A_{00}}$	1.4436	1.2349	9.24E-06	4.38E-05
$\boldsymbol{A_{10}}$	-0.0209	-0.4093	4.54E-04	2.16E-03
$\boldsymbol{A_{01}}$	-0.1014	-0.4812	7.30E-05	3.46E-04
$\boldsymbol{A_{20}}$	0.0009	0.0042	4.44E-04	2.11E-03
$\boldsymbol{A_{11}}$	0.0011	0.0052	5.81E-04	2.76E-03
$\boldsymbol{A_{02}}$	0.0001	0.0005	1.28E-04	6.07E-04

For both ternary systems, the refractive index and density data with the compositions were subjected to optimization to obtain the respective calibration curve functions (Eqs. 6 and 7), by minimizing the object function OF (Eq. S1) using the Levenberg-Marquardt algorithm. The coefficients of the calibration curves for the ηD and ρ with the compositions are shown in Tables S2 and S4 for the methyl palmitate + methanol + glycerol and ethyl palmitate + ethanol + glycerol systems, respectively, at 333.15 K.

$$\mathbf{FO} = \left|\boldsymbol{\eta}\mathbf{D}_{\mathbf{calibration}} - \boldsymbol{\eta}\mathbf{D}_{\mathbf{experimental}}\right| + \left|\boldsymbol{\rho}_{\mathbf{calibration}} - \boldsymbol{\rho}_{\mathbf{experimental}}\right| \qquad (\mathbf{S1})$$

Table S5. Refractive indices and densities (318.15 K) for the methyl palmitate (1) + methanol (2) + glycerol (3) systems (VLLE)

x_1	x_2	x_3	η_D	ρ (g.cm^{-3})
Palmitate phase				
0.5004	0.4890	0.0106	1.4322	0.9401
0.4518	0.5373	0.0110	1.4323	0.9392
0.4147	0.5689	0.0165	1.4322	0.9389
0.3799	0.6016	0.0185	1.4321	0.9358
0.3479	0.6323	0.0198	1.4320	0.9371
0.3194	0.6606	0.0200	1.4319	0.9378
0.2943	0.6867	0.0190	1.4318	0.9379
0.2711	0.7101	0.0188	1.4317	0.9377
0.2230	0.7593	0.0176	1.4314	0.9356
0.2075	0.7769	0.0157	1.4313	0.9345
0.1926	0.7920	0.0153	1.4312	0.9332
0.1788	0.8057	0.0155	1.4311	0.9318
0.1666	0.8189	0.0145	1.4310	0.9304
0.1497	0.8362	0.0141	1.4309	0.9282
0.1392	0.8462	0.0146	1.4308	0.9268
0.1299	0.8562	0.0139	1.4307	0.9256
0.1086	0.8773	0.0141	1.4305	0.9227
0.1012	0.8854	0.0134	1.4304	0.9218
0.0940	0.8928	0.0131	1.4304	0.9210
0.0838	0.9034	0.0128	1.4302	0.9199
0.0778	0.9107	0.0115	1.4302	0.9194
0.0763	0.9124	0.0113	1.4301	0.9193
0.0635	0.9268	0.0097	1.4299	0.9183
0.0597	0.9315	0.0087	1.4298	0.9180
0.0537	0.9388	0.0075	1.4296	0.9176
0.0512	0.9414	0.0075	1.4295	0.9173
0.0489	0.9440	0.0071	1.4294	0.9171
0.0446	0.9493	0.0061	1.4291	0.9165
0.0424	0.9516	0.0060	1.4290	0.9161
0.0403	0.9539	0.0059	1.4289	0.9157
0.0365	0.9588	0.0048	1.4286	0.9148
0.0355	0.9598	0.0047	1.4285	0.9145
0.0345	0.9610	0.0045	1.4284	0.9143
0.0308	0.9652	0.0040	1.4280	0.9129
0.0299	0.9662	0.0039	1.4279	0.9125
0.0291	0.9673	0.0036	1.4278	0.9121
0.0273	0.9693	0.0034	1.4276	0.9111
0.0256	0.9711	0.0033	1.4273	0.9100
0.0239	0.9732	0.0029	1.4271	0.9089
0.0223	0.9750	0.0027	1.4268	0.9076
0.0206	0.9767	0.0027	1.4265	0.9061

0.0198	0.9775	0.0026	1.4263	0.9053
0.0189	0.9782	0.0028	1.4261	0.9043
0.0188	0.9788	0.0024	1.4261	0.9041
0.0174	0.9800	0.0026	1.4258	0.9025
0.0168	0.9810	0.0022	1.4256	0.9017
0.0159	0.9817	0.0024	1.4254	0.9006
0.0144	0.9833	0.0022	1.4250	0.8984
0.0143	0.9839	0.0019	1.4250	0.8981
0.0138	0.9843	0.0019	1.4249	0.8974
0.0123	0.9858	0.0019	1.4244	0.8948
0.0116	0.9867	0.0017	1.4242	0.8936
0.0103	0.9883	0.0015	1.4238	0.8908
0.0096	0.9890	0.0014	1.4235	0.8893
0.0086	0.9901	0.0013	1.4231	0.8869
0.0076	0.9913	0.0011	1.4228	0.8845
0.0069	0.9921	0.0010	1.4225	0.8828
0.0060	0.9929	0.0011	1.4221	0.8803
0.0056	0.9944	0.0000	1.4218	0.8607
		Glycerol phase		
0.0000	0.2812	0.7188	1.4632	1.3263
0.0000	0.4029	0.5971	1.4628	1.3261
0.0000	0.4938	0.5062	1.4626	1.3250
0.0000	0.5820	0.4180	1.4581	1.3181
0.0000	0.6481	0.3519	1.4556	1.3126
0.0000	0.6983	0.3017	1.4532	1.3065
0.0000	0.7423	0.2577	1.4524	1.3007
0.0000	0.7766	0.2234	1.4506	1.2953
0.0000	0.8054	0.1946	1.4484	1.2900
0.0000	0.8242	0.1758	1.4442	1.2884
0.0000	0.8424	0.1576	1.4407	1.2871
0.0000	0.8577	0.1423	1.4374	1.2859
0.0000	0.8707	0.1293	1.4349	1.2840
0.0000	0.8834	0.1166	1.4323	1.2643
0.0000	0.8955	0.1045	1.4304	1.2418
0.0000	0.9061	0.0939	1.4286	1.2306
0.0000	0.9165	0.0835	1.4260	1.2187
0.0000	0.9255	0.0745	1.4246	1.2040
0.0000	0.9355	0.0645	1.4133	1.1542
0.0000	0.9442	0.0558	1.4026	1.1120
0.0000	0.9563	0.0437	1.3928	1.1037

Table S6. Coefficients of the calibration curves (VLLE) for the methyl palmitate + methanol + glycerol system (318.15 K and 101.3 kPa)

Coeficients	ηD	ϱ (g.cm^{-3})	Std error (η_D)	Std error (ϱ)
		Palmitate phase		
A_{00}	1.4223	0.8821	0.0003	0.0020
A_{10}	0.0103	0.2039	0.0012	0,0971
A_{01}	1.4104	6.4001	0.0127	0.0983
A_{20}	-0.0543	-0.5685	0.0224	0.0175
A_{11}	2.1516	13.6163	0.0483	0.0756
A_{02}	-78.3651	-45.7672	0.0872	0.0069
		Glycerol phase		
A_{00}	1.4010	1.1335	0.0046	0.0222
A_{10}	-8.9389	-8.2397	0.0026	0.0013
A_{01}	0.2633	0.9254	0,0366	0.0177
A_{20}	1.0000	1.0000	0.0000	0.0000
A_{11}	1.0000	1.0000	0.0000	0.0000
A_{02}	-0.2596	-0.9781	0.0512	0.0247

Table S7. Refractive indices and densities for the ethyl palmitate (1) + ethanol (2) + glycerol (3) system at 318.15 K (VLLE)

x_1	x_2	x_3	ηD	ϱ (g.cm^{-3})
0.5617	0.4090	0.0293	1.4161	0.8282
0.5144	0.4562	0.0294	1.4145	0.8274
0.4771	0.4878	0.0351	1.4133	0.8267
0.4432	0.5202	0.0367	1.4118	0.8265
0.4087	0.5533	0.0380	1.4091	0.8261
0.3786	0.5838	0.0376	1.4085	0.8255
0.3519	0.6122	0.0359	1.4062	0.8231
0.3267	0.6377	0.0356	1.4047	0.8222
0.3047	0.6625	0.0328	1.4038	0.8209
0.2996	0.6714	0.0290	1.4023	0.8197
0.2729	0.6938	0.0333	1.4008	0.8194
0.2562	0.7149	0.0290	1.3988	0.8175
0.2385	0.7319	0.0296	1.3982	0.8153
0.2224	0.7482	0.0294	1.3958	0.8144
0.2081	0.7639	0.0280	1.3949	0.8126
0.2063	0.7702	0.0235	1.3937	0.8103
0.2035	0.7743	0.0222	1.3931	0.8102
0.1881	0.7848	0.0271	1.3917	0.8098
0.1746	0.7978	0.0276	1.3901	0.8094
0.1645	0.8092	0.0264	1.3895	0.8081
0.1519	0.8306	0.0175	1.3869	0.8053
0.1381	0.8356	0.0263	1.3856	0.8042
0.1291	0.8458	0.0251	1.3839	0.8033
0.1203	0.8548	0.0249	1.3814	0.8021
0.1148	0.8667	0.0185	1.3807	0.8013
0.1071	0.8676	0.0254	1.3791	0.8006
0.1000	0.8780	0.0220	1.3782	0.7995
0.0980	0.8801	0.0219	1.3776	0.7971
0.0902	0.8890	0.0208	1.3758	0.7953
0.0851	0.8944	0.0205	1.3747	0.7948
0.0814	0.8991	0.0195	1.3738	0.7937
0.0795	0.9017	0.0187	1.3721	0.7928
0.0770	0.9046	0.0184	1.3717	0.7921
0.0731	0.9115	0.0153	1.3711	0.7897
0.0694	0.9144	0.0162	1.3693	0.7891
0.0661	0.9179	0.0160	1.3688	0.7884
0.0632	0.9214	0.0154	1.3684	0.7873
0.0606	0.9275	0.0119	1.3666	0.7854
0.0579	0.9280	0.0141	1.3662	0.7845
0.0549	0.9312	0.0139	1.3653	0.7839
0.0521	0.9343	0.0137	1.3643	0.7833
0.0500	0.9346	0.0154	1.3639	0.7824
0.0471	0.9403	0.0125	1.3633	0.7809
0.0463	0.9425	0.0112	1.3629	0.7797
0.0447	0.9451	0.0102	1.3615	0.7781
0.0427	0.9482	0.0091	1.3612	0.7775
0.0387	0.9508	0.0105	1.3597	0.7769

0.0379	0.9520	0.0101	1.3594	0.7765
0.0352	0.9547	0.0100	1.3583	0.7754
0.0345	0.9570	0.0085	1.3581	0.7746
0.0307	0.9596	0.0098	1.3569	0.7741
0.0286	0.9623	0.0091	1.3558	0.7733
0.0282	0.9645	0.0073	1.3557	0.7719
0.0263	0.9644	0.0093	1.3551	0.7717
0.0253	0.9652	0.0095	1.3548	0.7715
0.0242	0.9668	0.0090	1.3544	0.7712
0.0241	0.9676	0.0084	1.3541	0.7705
0.0232	0.9696	0.0072	1.3533	0.7691
0.0219	0.9682	0.0099	1.3531	0.7688
0.0207	0.9713	0.0080	1.3528	0.7686
0.0202	0.9715	0.0082	1.3526	0.7685
0.0194	0.9738	0.0068	1.3521	0.7681
0.0181	0.9746	0.0073	1.3516	0.7678
0.0174	0.9753	0.0073	1.3514	0.7676
0.0169	0.9758	0.0073	1.3511	0.7673
0.0164	0.9767	0.0070	1.3508	0.7669
0.0155	0.9764	0.0081	1.3506	0.7667
0.0149	0.9779	0.0072	1.3503	0.7665
0.0146	0.9785	0.0069	1.3502	0.7662
0.0141	0.9787	0.0072	1.3499	0.7661
0.0136	0.9797	0.0066	1.3496	0.7656
0.0131	0.9797	0.0072	1.3495	0.7655
0.0127	0.9807	0.0066	1.3492	0.7652
0.0122	0.9809	0.0069	1.3488	0.7647
0.0105	0.9830	0.0065	1.3478	0.7637
0.0092	0.9846	0.0062	1.3472	0.7635
0.0086	0.9854	0.0059	1.3471	0.7631
0.0079	0.9859	0.0062	1.3469	0.7626
0.0075	0.9874	0.0051	1.3468	0.7618
0.0071	0.9867	0.0062	1.3467	0.7617
0.0068	0.9873	0.0059	1.3464	0.7613
0.0065	0.9886	0.0048	1.3461	0.7606

Table S8. Coefficients of the calibration curves (VLLE) for the ethyl palmitate + ethanol + glycerol system at 318.15 K.

Coeficients	η_D	ρ (g.cm^{-3})	Std error ηD	Std error ρ
A_{00}	1.3412	0.7529	0.0005	0.0009
A_{10}	0.0833	0.0890	0.0016	0.0030
A_{01}	0.1726	0.5141	0.0542	0.1013
A_{20}	-0.0015	-0.0167	0.0016	0.0030
A_{11}	0.0079	0.2213	0.0345	0.0644
A_{02}	-2.3376	-7.0949	0.7541	0.4096

Table S9. Constants from the Antoine equation used to calculate the saturation pressure.

Component	A	B	C	D	E
Methyl palmitate[a]	6.247	3709.672	-1.062	---	---
Ethyl palmitate[b]	13.561	5363.871	43.329	---	---
Metanol[c]	82.718	-6904.5	-8.8622	$7.4664*10^{-06}$	2
Ethanol[c]	73.304	-7122.3	-7.1424	$2.8853*10^{-06}$	2
Glycerol[c]	99.986	-13808.0	-10.088	$3.5712*10^{-19}$	6

[a] P^{SAT} (bar) = exp [A – B/(T + C)], T in K [27]; [b] P^{SAT} (Pa) = exp [A – B/(T + C)], T in K [28]; [c] P^{SAT} (Pa) = exp [A + B/T + C*ln(T) + D*T^E], T in K [29]
